# Supplementary material for: Derivation of correlation dimension from spatial autocorrelation functions
Source: PLoS One. 2024 May 31;19(5):e0303212. doi: 10.1371/journal.pone.0303212 (PMC11142504; doi:10.1371/journal.pone.0303212)
Supplement: S1 File — This file provides a detailed derivation process for the relationship between spatial correlation dimension and spatial autocorrelation function based on Moran’s I. (DOCX) [file pone.0303212.s001.docx]

### Mathematical Derivation Process

A spatial proximity matrix, **V**, which is a spatial distance matrix or a spatial relation matrix, can be converted into a contiguity matrix as follows

$\mathbf{V}=\left[v_{ij}\right]_{N\times N}=\left[\begin{matrix} v_{11} & v_{12} & \cdots& v_{1N} \\ v_{21} & v_{22} & \cdots& v_{2N} \\ \vdots& \vdots& \ddots& \vdots\\ v_{N1} & v_{N2} & \cdots& v_{NN} \end{matrix}\right]$. (A1)

The spatial contiguity can be defined by a relative step function as below

$v_{ij}(r)=\left\{\begin{matrix} 1,\text{ 0<}d_{ij}\leq r \\ 0,\text{ }d_{ij}>r \end{matrix}\right.$ , (A2)

where *d_ij_* refers to the distance between locations *i* and *j*, *r* denotes a variable distance threshold. For the diagonal elements (*i*=*j*), if *d_ii_*=0 suggests *v_ii_*(*r*)=0, then we will have

$\mathbf{M}(r)=\left[v_{ij}(r)\right]_{N\times N}=\left[\begin{matrix} 0 & v_{12}(r) & \cdots& v_{1N}(r) \\ v_{21}(r) & 0 & \cdots& v_{2N}(r) \\ \vdots& \vdots& \ddots& \vdots\\ v_{N1}(r) & v_{N2}(r) & \cdots& 0 \end{matrix}\right]$. (A3)

This is one basis for conventional spatial autocorrelation analysis. On the other, for *i*=*j*, if *d_ii_*=0 suggests *v_ii_*(*r*)=1, then we will have

$\mathbf{M}^{*}(r)=\left[v_{ij}(r)\right]_{N\times N}=\left[\begin{matrix} 1 & v_{12}(r) & \cdots& v_{1N}(r) \\ v_{21}(r) & 1 & \cdots& v_{2N}(r) \\ \vdots& \vdots& \ddots& \vdots\\ v_{N1}(r) & v_{N2}(r) & \cdots& 1 \end{matrix}\right]$. (A4)

This will be used to make generalized spatial autocorrelation analysis. Obviously, the difference between **M**^*^(*r*) and **M**(*r*) is a unit matrix **E**, that is

$\mathbf{M}^{*}(r)-\mathbf{M}(r)=\mathbf{E}$. (A5)

The sum of the elements in the contiguity matrix is as follows

$T(r)=\sum_{i=1}^{n} \sum_{j=1}^{n} v_{ij}(r)=\left\{\begin{matrix} M_{0}(r),\text{ }v_{ii}=0 \\ M_{0}^{*}(r),\text{ }v_{ii}=1 \end{matrix}\right.$. (A6)

Define an ones vector **o**=[1, 1, …, 1]^T^, we have

$M_{0}(r)=\mathbf{o}^{\text{T}}\mathbf{M}(r)\mathbf{o}$,$M_{0}^{*}(r)=\mathbf{o}^{\text{T}}\mathbf{M}^{*}(r)\mathbf{o}$. (A7)

Apparently, *N*=*n*=**o**^T^**Eo**. Thus the number of non-zero elements in the matrix **M**(*r*) is

$N(r)=M_{0}^{*}(r)=M_{0}(r)+N=\mathbf{o}^{\text{T}}\mathbf{M}^{*}(r)\mathbf{o}$. (A8)

According to equation (7), *N*(*r*) is just the correlation number of cities. In order to unitize the spatial contiguity matrix, define

$\frac{v_{ij}(r)}{T(r)}=\frac{v_{ij}(r)}{\sum_{i=1}^{n} \sum_{j=1}^{n} v_{ij}(r)}=\left\{\begin{matrix} w_{ij}(r),\text{ }v_{ii}(r)=0 \\ w_{ij}^{*}(r),\text{ }v_{ii}(r)=1 \end{matrix}\right.$. (A9)

Thus we have

$\mathbf{W}(r)=\frac{\mathbf{M}(r)}{M_{0}(r)}=\left[w_{ij}(r)\right]_{n\times n}$, $\mathbf{W}^{*}(r)=\frac{\mathbf{M}^{*}(r)}{M_{0}^{*}(r)}=\left[w_{ij}^{*}(r)\right]_{n\times n}$. (A10)

With the preparation of the above definitions and symbolic system, we can define the spatial autocorrelation function. Based on standardized size vector **z** and global unitized spatial weight matrix **W**, Moran’s index of spatial autocorrelation can be expressed as (Chen, 2013a)

$I=\mathbf{z}^{\text{T}}\mathbf{Wz}$. (A11)

Replacing the determined unitized spatial weight matrix **W** by the variable unitized spatial weight matrix **W**(*r*) yields

$I(r)=\mathbf{z}^{\text{T}}\mathbf{W}(r)\mathbf{z}$, (A12)

which is a spatial autocorrelation function of displacement based on Moran’s index (Chen, 2021). If we consider the zero-lag self-correlation of geographical elements, Moran’s index can be generalized to the following form

$I^{*}(r)=\mathbf{z}^{\text{T}}\mathbf{W}^{*}(r)\mathbf{z}$. (A13)

In the spatial weight matrix **W**^*^(*r*), the values of the diagonal elements are 1. The matrix expression of the spatial autocorrelation function based on Moran’s index can be decomposed as

$I(r)=\mathbf{z}^{\text{T}}(\frac{\mathbf{M}(r)}{M_{0}(r)})\mathbf{z}=\mathbf{z}^{\text{T}}(\frac{\mathbf{M}^{*}(r)}{M_{0}(r)}-\frac{\mathbf{E}}{M_{0}(r)})\mathbf{z}=\frac{1}{M_{0}(r)}\text{(}\mathbf{z}^{\text{T}}\mathbf{M}^{*}(r)\mathbf{z}-N)$, (A14)

in which the total number of all elements in a given geographical system can be expressed as

$N=\mathbf{z}^{\text{T}}\mathbf{Ez}=\mathbf{z}^{\text{T}}\mathbf{z}$. (A15)

Thus, equation (A14) can be rewritten as

$M_{0}(r)I(r)=\mathbf{z}^{\text{T}}\mathbf{M}^{*}(r)\mathbf{z}-N$. (A16)

The two sides of equation (A16) divided by the correlation number *N*(*r*) at the same time yields

$\frac{M_{0}(r)I(r)}{N(r)}=\mathbf{z}^{\text{T}}\frac{\mathbf{M}^{*}(r)}{M_{0}^{*}(r)}\mathbf{z}-\frac{N}{N(r)}=\mathbf{z}^{\text{T}}\mathbf{W}^{*}(r)\mathbf{z}-\frac{N}{N(r)}=I^{*}(r)-\frac{N}{N(r)}$. (A17)

This suggests that the autocorrelation function based on the generalized Moran’s index can be decomposed as follows

$I^{*}(r)=\mathbf{z}^{\text{T}}\mathbf{W}^{*}(r)\mathbf{z}=\frac{N}{N(r)}+\frac{M_{0}(r)I(r)}{N(r)}=\frac{\mathbf{z}^{\text{T}}\mathbf{Ez}+\mathbf{z}^{\text{T}}\mathbf{M}(r)\mathbf{z}}{\mathbf{o}^{\text{T}}\mathbf{M}^{*}(r)\mathbf{o}}$. (A18)

From equation (A17) it follows

$I^{*}(r)-\frac{M_{0}(r)I(r)}{M_{0}(r)+N}=I^{*}(r)-\frac{I(r)}{1+N/M_{0}(r)}=\frac{N}{N(r)}$. (A19)

Substituting equation (8) into equation (A19) yields

$I^{*}(r)-\frac{I(r)}{1+N/M_{0}(r)}=\frac{N}{N(r)}=\frac{N}{N_{1}}r^{-D_{c}}$, (A20)

which gives the mathematical relationships between the spatial autocorrelation function, *I*(*r*), the generalized autocorrelation function, *I*^*^(*r*), and the spatial correlation dimension, *D*_c_. Considering equation (4), *C*(*r*)=*N*(*r*)/*N*^2^, we have a spatial correlation equation such as

$\frac{1}{C(r)}=NI^{*}(r)-\frac{N}{1+N/M_{0}(r)}I(r)=\frac{N^{2}}{N_{1}}r^{-D_{c}}$. (A21)

With the increase of *r*, *N*/*M*_0_(*r*) approaches 0. Thus, for large spatial datasets, we have an approximate expression as below:

$\Delta I(r)=I^{*}(r)-I(r)\approx\frac{N}{N(r)}=\frac{1}{NC(r)}=\frac{N}{N_{1}}r^{-D_{c}}=\frac{1}{NC_{1}}r^{-D_{c}}$, (A22)

where ∆*I*(*r*) denotes the difference between *I*^*^(*r*) and *I*(*r*), and the parameter *C*_1_=*N*_1_/*N*^2^.
