# Supplementary material for: Derivation of correlation dimension from spatial autocorrelation functions
Source: PLoS One. 2024 May 31;19(5):e0303212. doi: 10.1371/journal.pone.0303212 (PMC11142504; doi:10.1371/journal.pone.0303212)
Supplement: Attachment — Submitted filename: Response to reviewers-2024-02-29.docx [file pone.0303212.s004.docx]

## Response to Editor and Reviewers

### **Editor comment:**

**[Editor]** The paper's primary contribution lies in its ability to compute spatial correlation dimension using the standard spatial autocorrelation function. However, the manuscript lacks grammatical clarity and a coherent flow. In addition to editorial revisions, the authors should provide a clear description of how users can employ their method. It would be beneficial to include any R-function for implementation, along with brief instructions, as supplementary material.

It is unfortunate that many reviewers declined to review this manuscript, some even after agreeing to do so. To prevent unnecessary delays, the author must submit a revised version based on my assessment and one reviewer's comment. Since the submitted version lacked line numbers, it was difficult to locate the comments.

#### Specific comments.

**[Editor]** Abstract:

- Rewrite it with subheading: Background, Method, Result and Conclusion.

**[Reply]** The abstract has been revised. A structural abstract was provided now.

**[Editor]** Introduction:

- Review <https://doi.org/10.1016/j.spasta.2020.100454>

**[Reply]** In the section of Introduction, the paper about B-statistics has been cited in two places as follows.

“…and many other fields (Chen, 2013a; Ejigu and Wencheko, 2020).”

“Recently, a new spatial statistic called *B*-statistic, which is based on both geographical and covariate-proximity, was proposed for measuring spatio-environmental autocorrelation (Ejigu and Wencheko, 2020)”

**[Editor]** Theoretical models

- On page 6 (last line), correct *d_ij_*=0, is it to mean *d_ij_*>*r*.

**[Reply]** There are deficiencies in expression here, leading to misunderstandings. Actually, what is being discussed here is the diagonal elements. Now, the related expressions have been revised as below:

“For the diagonal elements (*i*=*j*), if *d_ii_*=0 suggests *v_ii_*(*r*)=0, then we will have…This is one basis for conventional spatial autocorrelation analysis.”

“On the other, for *i*=*j*, if *d_ii_*=0 suggests *v_ii_*(*r*)=1, then we will have…This will be used to make generalized spatial autocorrelation analysis.”

**[Editor]** - There are so many equations in the manuscript (above 45). Take many of the equation presented from page 7-10 to the appendix with additional details.

**[Reply]**Many equations have been transferred to appendixes.

**[Editor]** - In one paragraph clearly summarize the distinct features of your approach.

**[Reply]** Many equations have been transferred to appendixes.

**[Editor]** Empirical analysis

- Ident (to the left) the computational steps outlined from P.10 to P.11.

**[Reply]** The computational steps have been rearranged for identifying to the left.

**[Editor]** - Table 3 summarized different statistics. What about the B-statistics (<https://doi.org/10.1016/j.spasta.2020.100454>)

**[Reply]** I read the paper about B-statistics, it is very interesting. Unfortunately, I haven't figured out how to estimate the spatial correlation dimension based on *B*-statistics for the time being. Perhaps in the future, I will find a breakthrough. This was discussed in in the section of Discussion as one of shortcomings of this study.

**[Editor]**- How the parameters in Eqn 35 obtained?

**[Reply]** A sentence has been added below equation (35) to explain the parameter estimation method: “The parameter values of the model are estimated by double logarithmic linear regression based on the least squares calculation.”

**[Editor]**- Briefly summarize the results presented under Table 2.

**[Reply]** A paragraph has been added to the note below Table 2:

“The main model parameters appearing in this section were estimated by using the data in this table. The relationship between *r* and *N*(*r*) gives equation (19), the relationship between *r* and *N*^*^(*r*) gives equation (20), the relationship between *r* and 1/(*NC*(*r*)) gives equation (21), which suggests equation (22). ∆*I* is the difference between *I*(*r*) and *I*^*^(*r*). The relationships between *r* and ∆*I* give equations (23) and (24).”

**[Editor]**Discussion

- Explain the added value of the proposed method over Moran’s *I* and *B*-statistic.

Now, a paragraph has been added to the section of Discussion as follows:

“The shortcoming of this work lies in three respects. First, the spatial weight matrix is based on conventional concept, and special spatial separation factors have not been considered. The spatial autocorrelation function and corresponding correlation dimension rely heavily on the form, structure and types of spatial weight matrices. Generally speaking, weighting matrices for spatial autocorrelation analysis are constructed by means of the concept of spatial proximity or distance-decay functions. However, in some cases, two locations which are close geographically but separated by other factors such as lakes, rivers, mountains, and buildings may not be treated as near neighbors. A new finding is that weighting matrices based on combination of geographical proximity with covariate information may solve the problem, and *B*-statistics as a new spatial measure emerged as the times require (Ejigu and Wencheko, 2020). Unfortunately, how to use the *B*-statistic including covariate information to construct spatial autocorrelation function and estimate spatial correlation dimension is an outstanding issue.”

**[Ref.]** Ejigu BA, Wencheko E (2020). Introducing covariate dependent weighting matrices in fitting autoregressive models and measuring spatio-environmental autocorrelation. *Spatial Statistics*, 38: 100454

### **Reviewer #1**

**[Reviewer #1]** This study reveals the inherent association of fractal patterns with spatial autocorrelation processes. The overall idea of the article is reasonable, and the technical approach is relatively mature. However, there are also some shortcomings. The author should revise the paper carefully according to the comments.

**[Reply]** Thank you very much for accepting the review invitation of this article and providing constructive comments.

Specific Opinions:

**[Reviewer #1]** 1. The abstract of the article lacks data support. The results of the study are explained in detail in the abstract, but the presentation of specific experimental data is lacking. Authors are advised to revise the abstract and add a description of the data used.

**[Reply]** The abstract has been revised now.

Now, a paragraph was added to the section of Empirical Analysis to emphasize the relationship between models and data as below:

“The goal of this study is to establish a mathematical model that connects spatial autocorrelation and spatial scaling laws. A as Neumann (1961) once said: “The sciences do not try to explain, they hardly even try to interpret, they mainly make models.” In this sense, this work relies heavily on mathematical derivation and modeling rather than data analysis. After all, as Karlin (1983) pointed out: “The purpose of models is not to fit the data, but to sharpen the questions.” Despite this, it is significant to verify a modeling result by observational data. I am very much in favor of his viewpoint of Louf and Barthelemy (2014), who said: “The success of natural sciences lies in their great emphasis on the role of quantifiable data and their interplay with models. Data and models are both necessary for the progress of our understanding: data generate stylized facts and put constraints on models. Models on the other hand are essential to comprehend the processes at play and how the system works. If either is missing, our understanding and explanation of a phenomenon are questionable. This issue is very general, and affects all scientific domains, including the study of cities.”

**[Reviewer #1]** 2. Necessary citation information can enhance the reliability and accuracy of the article. In terms of literature citation, this article is mainly related to the older years. In order to reflect the research significance and value of this study, it is suggested that the authors should add citations of relevant articles in full-text journals in recent years.

**[Reply]** You are right. The origin of this research work is the measurement difficulties encountered in the teaching process of quantitative geography. In other words, the discovery of research questions is based on teaching rather than literature review. According to your constructive comments, I have searched for relevant literature in recent years as much as possible and cited them. If you find that I have overlooked some important relevant literature, please let me know them specifically.

As we know, the purpose of literature review in a paper is mainly twofold: firstly, to clarify the academic background of a research, and secondly, to provide reference for writing innovative points in the paper. After completing the manuscript, I conducted literature review through Google Scholar and Web of ISI. I have found that there are many articles that use spatial autocorrelation analysis to conduct empirical research, but the content of those papers has no clear relationship with the background and innovation of this study. Most of the theoretical and methodological studies on spatial autocorrelation analysis related to my paper were published before 2000. During the process of revising this article, based on your comments, I conducted another literature search, but the results were limited.

There is a plethora of literature, and a person's perspective is limited after all. I will be very appreciated if you have noticed any relevant literature and let me know.

Now, a number of references have been added to the reference list. See for example:

Bjørnstad ON, Falck W (2001). Nonparametric spatial covariance functions: Estimation and testing. *Environmental and Ecological Statistics*, 8: 53-70

De Knegt HJ, van Langevelde F, Coughenour MB, Skidmore AK, De Boer WF, Heitkönig IMA, Knox NM, Slotow R, van Der Waal C, Prins HHT (2010). Spatial autocorrelation and the scaling of species–environment relationships. *Ecology*, 91 (8): 2455–2465

Dray S (2011). A new perspective about Moran's coefficient: spatial auto-correlation as a linear regression problem. *Geographical Analysis*, 43(2): 127-141

Dray S, Legendre P, Peres-Neto PR (2006). Spatial modeling: a comprehensive framework for principal coordinate analysis of neighbor matrices (PCNM). *Ecological Modelling*, 196(3-4): 483-493

Dubin RA (1998). Spatial autocorrelation: A primer. *Journal of Housing Economics*, 7(4): 304-327

Ejigu BA, Wencheko E (2020). Introducing covariate dependent weighting matrices in fitting autoregressive models and measuring spatio-environmental autocorrelation. *Spatial Statistics*, 38: 100454

Geniaux G, Martinetti D (2018). A new method for dealing simultaneously with spatial autocorrelation and spatial heterogeneity in regression models. *Regional Science and Urban Economics*, 72: 74-85

Griffith DA (2019). Most neglected concepts in spatial statistics. *Stats*, 2019, 2(3): 388-415

Griffith DA, Chun YW (2016). Spatial autocorrelation and uncertainty associated with remotely-sensed data. *Remote Sensing*, 8(7): 535

Griffith DA, Morris ES, Thakar V (2016). Spatial autocorrelation and qualitative sampling: The case of snowball type sampling designs. *Annals of the American Association of Geographers*, 106(4): 773-787

Kim D (2021). Predicting the magnitude of residual spatial autocorrelation in geographical ecology. *Ecography*, 44(7): 1121-1130

Legendre P (1993). Spatial autocorrelation: Trouble or new paradigm? *Ecology*, 74(6): 1659-1673

Legendre P, Legendre L (1998). *Numerical Ecology* (2^nd^ edition). Amsterdam: Elsevier Science

Naimi B, Skidmore AK, Groen TA, Hamm NAS (2011). Spatial autocorrelation in predictors reduces the impact of positional uncertainty in occurrence data on species distribution modelling. *Journal of Biogeography*, 38(8): 1497-1509

Xiao YX, Gong P (2022). Removing spatial autocorrelation in urban scaling analysis. *Cities*, 124: 103600

**[Reviewer #1]** 3. The innovation point of the research is not sufficiently concise. The most outstanding innovation of this study is that it reveals the intrinsic relationship between fractal patterns and spatial autocorrelation processes. It is recommended to polish and revise the stiff parts of the creative point writing.

**[Reply]** The innovative points of this study are re-summarized as follows:

“Compared with the previous studies on spatial autocorrelation, this work is based on scaling law rather than characteristic scales. In literature, some studies involved the concept of spatial scaling, but do not delve into the essence of scaling laws (De Knegt *et al*, 2010). Some scholars attempt to lessen the influence of spatial autocorrelation in the process of conducting scaling analysis of urban hierarchies (Xiao and Gong, 2021). The novelty of this paper lies in revealing the mathematical relationships between spatial autocorrelation functions and spatial correlation dimension. First, the strict mathematical relation between Moran’s index set and spatial correlation dimension was derived. Thus, the common spatial analysis based on characteristic scale is associated with the spatial analysis based on scaling law. Second, the above-mentioned relation was generalized to Ceary’s coefficient and Getis-Ord’s index. This is helpful for developing a logic framework for spatial scaling analysis based on autocorrelation functions. Third, a complete case analysis was presented. This is useful for understanding spatial autocorrelation of Chinese cities. Fractal dimension suggests scaling law, and scaling invariance suggests nonlinear spatial autocorrelation rather than linear autocorrelation. The linear autocorrelation process is simple and can be described and analyzed using linear algebra and statistics. The nonlinear autocorrelation process is complex and requires the introduction of scaling analysis ideas and methods based on linear algebra and statistics to effectively describe and analyze it.”
